# Neuroprotective and Neurodegenerative Aspects of Coffee and Its Active Ingredients in View of Scientific Literature

**DOI:** 10.7759/cureus.9578

**Published:** 2020-08-05

**Authors:** Shehnaz Wasim, Vishal Kukkar, Vanessa M Awad, Sirisha Sakhamuru, Bilal Haider Malik

**Affiliations:** 1 Internal Medicine, California Institute of Behavioral Neurosciences and Psychology, Fairfield, USA; 2 Radiology, California Institute of Behavioral Neurosciences and Psychology, Fairfield, USA; 3 Internal Medicine/Family Medicine, California Institute of Behavioral Neurosciences and Psychology, Fairfield, USA

**Keywords:** coffee consumption, caffeine, cognitive decline, alzheimers disease, dementia, stroke, neurodegenerative disesase, parkinson's disease

## Abstract

Coffee and its components have several neuroprotective properties that lower the risk of cognitive decline and other neurodegenerative diseases. This study reviews the mechanisms by which coffee and its respective compounds affect the brain and its pathologies. Many epidemiological studies in this literature review have shown coffee to reduce the risk of developing dementia, stroke, and Alzheimer's disease. It may also have a positive impact on the disease course of amyotrophic lateral sclerosis, Parkinson's disease, and depression. The optimal benefits achieved from coffee in these pathologies rely on higher daily doses. Most of its effects are attributed to caffeine by the antagonism of adenosine receptors in the central nervous system; however, other coffee constituents like chlorogenic acids have also shown much promise in therapeutic value. Existing research considers coffee to have great potential, but additional studies are still needed to clarify the mechanisms and actual causal relationships in certain neuropathologies.

## Introduction and background

Coffee is one of the most popular beverages. On average, Americans drink about two cups of coffee everyday [1]. Its exports worldwide amounted to more than 11 million 60 kg bags in March 2020 alone, posing a significant impact on the health of its consumers on such a population scale; therefore, studies regarding its effects have peaked in recent years [2].

Coffee contains more than a thousand compounds, many of which have yet to be found [3]. Its bioactive components include the most widely known caffeine and others, like chlorogenic acids (CGAs), diterpenes, trigonelline, tryptophan alkaloids, and secondary metabolites that are a product of Maillard reactions called melanoidins [4]. These have anti-inflammatory, antioxidant, antifibrotic, antimicrobial, and anti-cancer properties that lead to its beneficial role in lowering all-cause mortality and improving endocrine, liver, gastrointestinal, cardiovascular diseases, cancers, and, to which our paper focuses on, neurocognitive function and neurodegenerative diseases [5-7]. 

Coffee undergoes a number of chemical reactions starting from the type of bean (Arabica or Robusta) used to the level of roasting and the method of preparation by brewing or grind setting [8-10]. This altogether will determine the final composition of the cup of coffee, and the bioavailability of ingredients will depend on each individual's metabolism [11]. 

Moderate coffee intake (e.g., two to four cups, which is 200-400 mg) in a day is associated with the highest benefits, while risks are relatively low [12].

This popular beverage has reduced the risk of numerous neurological disorders such as Parkinson's disease (PD), depression, and neurocognitive decline, especially seen in Alzheimer's disease (AD) [5]. The reason for its popularity is the short-term effects that include raising alertness and energy majorly due to caffeine inhibiting adenosine receptors in the brain; however, higher doses (400-800 mg at one time) can account for anxiety, jitteriness, insomnia, and tachycardia [13]. Apart from caffeine, there are many more effects on the brain associated with other potential therapeutic components of coffee [14].

Observational studies provide evidence of coffee having an association with a myriad of benefits over different diseases. Still, very few have shown causal relations especially in a dose-dependent manner; therefore, more long-term randomized controlled trials will be needed with effective methods to eliminate confounding factors such as smoking to understand the actual therapeutic value of not only coffee as a whole but also its separate components, each having specific effects of their own. This study summarizes the impact of coffee and its respective chemical compounds on neurocognitive function and other brain pathologies. It discusses the mechanism of action of its constituents and highlights the degree of importance of coffee according to neurological disorders by comparing them with different studies.

## Review

Bioactive compounds of coffee and their properties

The most important compounds in coffee include methylxanthines (caffeine, theophylline and theobromine), polyphenols (CGAs and their derivatives like caffeic acid and pyrocatechol), diterpenes comprising cafestol and kahweol, flavonoids (anthocyanin and catechins), lactones, trigonelline, nicotinic acid (vitamin B3), and many more micronutrients like potassium and magnesium [3,6,15]. All of these have many physiological effects, some overlapping with others. When coffee undergoes roasting, amino acids and carbohydrates react through a process called Maillard reactions that produce additional compounds like melanoidins and acrylamide [4,6]. Although little is known, caffeine and polyphenols currently have great neuroprotective properties [14].

The widely studied caffeine has antagonistic activity against adenosine receptors in the central nervous system and elsewhere in the body resulting in psychoactive effects [13]. There is also a notable increase in metabolism, diuresis, and blood pressure in an acute setting [16]. Although caffeine dependence may be questionable as it has been mentioned, studies have shown moderate consumption has not increased dopaminergic transmission in nucleus accumbens, which is a characteristic finding for drugs of dependence [17,18]. Nucleus accumbens is the site of the brain that involves the "reward circuit" and is an essential component of the mesolimbic pathway. When this site is stimulated, it releases dopamine that is known for rewarding experiences that are implicated in drug addiction. In patients with headaches and migraines, caffeine may also increase the potency of pain medications and is proven therapeutic [13]. It has decreased the overall risk of developing stroke and neurodegenerative diseases [5]. It is also listed amongst WHO's 2019 list of essential medicines for use in apnea [19,20]. Although it covers innumerable benefits, caffeine intake is also associated with adverse effects in higher doses that are related to jitteriness, palpitations, insomnia, and anxiety [13]. This is due to the short-term effects that raise the sympathetic drive in humans, and thus aggravate conditions related to stress and anxiety. In a systematic review by Vilarim et al., an association of caffeine and panic disorder is indicated [21]. Caffeine is also found in other dietary sources, as shown by (Table 1) [22]. A general trend may be noticed in the table that coffee and its subtypes have the most caffeine except energy drinks.

**Table 1:** Sources of caffeine in the diet.

Sources of caffeine	Mean concentration
Espresso	60 mg/30 mL
Decaffeinated coffee	3 mg/125 mL
Filtered coffee	85 mg/125 mL
Instant coffee	65 mg/125 mL
Tea (leaves or bag)	32 mg/150 mL
Iced tea	20 mg/330 mL
Caffeinated soft drinks	39 mg/330 mL
Sugar-free soft drinks	41 mg/330 mL
Energy drinks	80 mg/330 mL
Hot chocolate	4 mg/150 mL
Chocolate bar	20 mg/30 g
Dark chocolate	60 mg/30 g
Milk chocolate	6 mg/30 g

Many studies have also shown coffee to attenuate inflammatory chemokines, such as interleukin 6, tumor necrosis factor-α, interferon-γ, and transforming growth factor-β. The main contributors being diterpenes, caffeine, trigonelline, and CGAs [23]. Caffeine itself demonstrates both anti-inflammatory and proinflammatory activity, as interpreted by Paiva et al. [24]. These benefits might play a crucial part in inflammatory brain disorders such as autoimmune diseases like multiple sclerosis.

Diterpenes like cafestol and kahweol exhibit important antioxidant and chemoprotective properties but also have raised serum cholesterol levels [25,26]. Antioxidant properties of chemical compounds generally eliminate free radicals in the human body. Free radicals, when in excess, cause oxidative stress leading to DNA damage and cell death. This further increases the risk of diseases like cancer. Trigonelline, along with melanoidins, also have antioxidant effects by similar mechanisms [27,28]. CGAs share not only antioxidant characteristics but also anti-inflammatory effects by inhibiting the expression of cytokine genes and modulating inflammatory nuclear factor kappa-light-chain-enhancer of activated B cells (NF-κB) activation and is associated with regeneration of neurons contributing to neuroplasticity [13,15]. 

Neurocognitive decline, dementia, and AD

Age-related cognitive decline is a growing issue in the elderly population [29]. Its progression to dementia is a continuous, irreversible process with treatment options that are very limited; therefore, reducing risk factors for the development of cognitive impairment is paramount. Caffeine attributes to most cognitive benefits. There are mixed studies depicting the positive association of decaffeinated coffee compared to caffeinated coffee affecting cognitive performance [30]. A recent publication by Dong et al. concluded from the National Health and Nutrition Examination Survey (NHANES), which was conducted by Centers for Disease Control and Prevention (CDC), that coffee, caffeinated coffee, and caffeine were associated with cognitive performance while decaffeinated coffee was not [30].

Moreover, studies also show this association in a dose-dependent manner, a greater number of coffee cups lower the risk of cognitive impairment, dementia, and AD [31]. An observational meta-analysis did observe positive effects of coffee (caffeine) on cognitive disorders with a relative risk (RR) of 0.84 (95% CI: 0.72-0.99, I2 = 42.6%) but then again another observational meta-analysis did not find such association [32,33]. The mechanism by which this occurs remains unclear. A study suggested it acts through cholinergic pathways that specifically enhance memory in humans [34]. Various animal studies show how caffeine enhances memory as well [35,36]. An overview of studies discussing coffee/caffeine intake with cognitive impairment and their results are shown in Table 2.

**Table 2:** Articles published demonstrating association of coffee/caffeine consumption with cognitive impairment. CI, confidence interval; RR, relative risk; OR, odds ratio; HR, hazard ratio.

Authors (reference)	Year of publication range	Type of study	Number of participants	Parameter used with 95% CI	Association found (yes/no)
Dong X et al. [30]	2011-2014	Meta-analysis	2,513	OR: 0.56 (0.35-0.89)	Yes
Wu L et al. [31]	Inception-2016	Meta-analysis	34,282	RR: 0.82 (0.71-0.94)	Yes
Santos C et al. [32]	1989-2009	Systematic review/meta-analysis	-	RR: 0.84 (0.72-0.99)	Yes
KIm YS et al. [33]	Inception-2014	Meta-analysis	31,479	OR/RR: 0.83 (0.70-0.98)	No
Solfrizzi V et al. [39]	-	Longitudinal study	1,445	HR: 0.26 (0.03-2.11)	no

**Figure 1:**
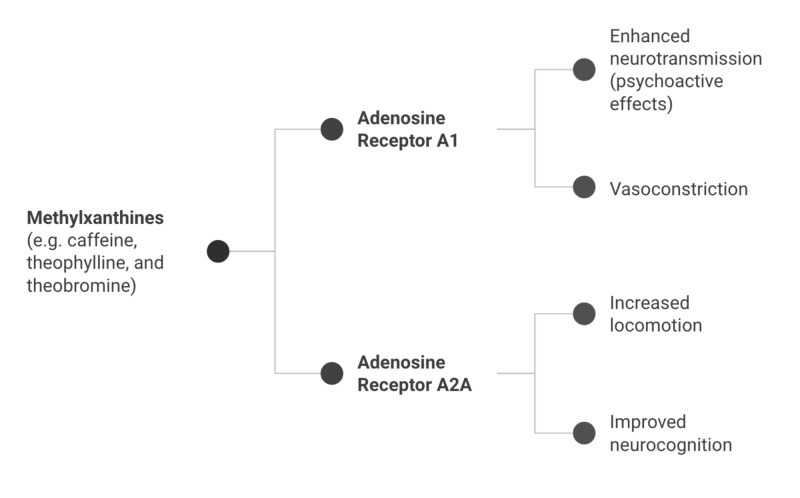
Effects of adenosine antagonism by methylxanthines in the brain.

AD is the most common cause of dementia [37]. Several studies have suggested that coffee is associated with reduced risks of developing AD [16]. This could be due to the effects of caffeine and CGAs on the adenosine receptors that ultimately play a role in preventing toxic β-amyloid peptide deposits in the brain [37]. This mechanism can be very specific to the extent and type of adenosine receptors involved because animal models with AD observed the neuroprotective potential of caffeine due to its action on A2AR rather than A1 receptors [38]. This indicates that adequate adenosinergic activation is needed for normal memory function and that an over- or underactivation can lead to memory impairment [39]. This further explains why a recent study states coffee intake of ≥2 cups/day was significantly associated with a lower Aβ positivity compared to lower coffee drinkers (<2 cups/day), resulting in decreased cerebral amyloid deposition [40]. As the benefits of coffee on these disorders are much attributed to methylxanthines (i.e., caffeine), Figure 1 illustrates how inhibiting the subtypes of adenosine receptors can have different effects on them. Adenosine A1 receptors enhance the release of neurotransmitters, while A2A receptors focus on improving neurocognitive function and neuroplasticity. This can be explored in greater depth as a pharmacological approach for such disease processes in the future.

Parkinson's disease 

Many epidemiological studies have suggested an association of coffee with the development of PD, while some may contradict with others [5,13,16,25,41]. The neuroprotective properties seen are attributed to caffeine and CGSs [25,37]. Again, there is a dose-relationship pattern that is observed in PD to achieve the most benefit from coffee being about 3 cups/day [42]. Coffee not only reduces the risk of PD but in a prospective study (n=16) appeared to improve "total akinesia" type of freezing on gait but for a limited number of months after which the patients developed tolerance. A two-week caffeine withdrawal restored these effects [43]. These potential beneficial effects of coffee (caffeine/CGAs) can be due to adenosine receptor antagonism in dopamine-rich areas of the brain that augment its release and enhance transmission [13,25]. A study in mice demonstrated neuroprotection on 1-methyl-4-phenyl-1,2,3,6 tetra-hydropyridine (MPTP) model of PD by virtue of A2AR activity [44]. The local antioxidant property of CGAs aids in neurogenesis and together with pyrogallol, trigonelline, catechol, 5-hydroxytryptamides, and N-methylpyridinium exhibits similar effects by increasing calcium signaling and dopamine release in the central nervous system [13,41]. Another explanation is that coffee raises the number of bifidobacteria that are associated with mitigating local inflammatory response, diminishing procarcinogenic processes and lower misfolding rates of α-synuclein in the enteric nervous system, therefore reducing the risk of PD by decreasing dissemination of the protein to the brain [45]. There are very few studies that have looked into this avenue and require further investigation to better understand the mechanism. It is of note that the strongest association among all neurodegenerative diseases has been indicated in caffeine consumption with PD incidence [46]. 

Stroke 

Several studies mention the association of coffee with the risk of stroke [5,6,13]. Patil et al. discusses many epidemiological studies regarding high coffee consumption, one being associated with a lower risk of total stroke, cerebral infarction, and subarachnoid hemorrhage but not intracerebral hemorrhage. This was after other risk factor adjustments were made [16]. A large meta-analysis of 36 cohort studies comprising 36,352 cases of cardiovascular diseases including stroke reported a 5% decrease in relative risk of stroke by a median consumption of 5 cups daily and 15% for 3.5 cups compared with a median consumption of zero [47]. Most prospective studies support a weak inverse proportionality between moderate coffee consumption and stroke risk [48]. Further placebo studies are needed to clarify the mechanisms and to elucidate a plausible causal relationship. Lifetime coffee intake may lead to late life cerebral white matter hyperintensities in cognitively normal elderly individuals, which signify cerebral hypoperfusion [49]. This might be attributed to the prolonged exposure of caffeine that results in vasoconstriction and decreases cerebral blood flow over time. It was seen in a Korean longitudinal study with women more affected than men, which can be explained by certain hormones (estradiol) that decrease the clearance of caffeine in women [49]. This gender disparity should also be taken into account when studies are being done. 

Others

Several studies discuss the implications of coffee in other diseases. Its role in depression has also been quite promising [14,37]. Nehlig mentions the effects of caffeine in elevating mood and reducing depression, contributing to the psychoactive properties of coffee [13]. Many people take coffee to drive sleep away and to prevent the drowsiness associated with the postprandial alkaline tide. A study by George et al. states that there is no clear evidence of caffeine consumption with sleep problems; however, a more recent study found that high lifetime coffee consumption decreases the overall pineal gland volume and this might disturb the quality of sleep later on in life [12,50]. In the management of many cases, coffee is to be avoided in the evening for people with sleep problems.

In a few experimental studies, caffeine showed a positive impact on the course of multiple sclerosis rather than affecting the development of the disease. On the other hand, no such benefits were observed in clinical and experimental studies on amyotrophic lateral sclerosis [50]. Other than this, there are many more benefits of coffee seen that include a better prognosis of patients with traumatic brain injury. The correlation in these patients was with higher cerebral spinal fluid caffeine levels [46].

## Conclusions

Coffee may reduce the overall risk of neurocognitive decline and play a beneficial role in other neurodegenerative diseases when consumed in moderate amounts. It is associated with very few harmful effects only in high doses. The exact mechanisms by which each component affects the brain still need to be further investigated on humans. Each compound and its effects must be studied separately as they have their own unique properties that can have different purposes according to the type of disease. 

The vast majority of studies discuss the association of coffee with different health outcomes but no causal relationship is identified. This is primarily due to the presence of observational studies that provide low quality evidence. Therefore we call upon randomized controlled trials to study coffee consumption in a dose-dependent manner within healthy and patient populations. Furthermore, it is essential to remove confounders and take into account the physiologic parameters and genetic variations that could alter conclusions drawn from studies by categorization. This way we may raise the statistical power of studies and better understand the potential pharmacological role of coffee in various disease processes. 
